# Knocking-out the brassinosteroid biosynthesis genes *DWARF1* or *DWARF4* produces upright canopy architecture in wheat

**DOI:** 10.1007/s00122-026-05418-z

**Published:** 2026-10-24

**Authors:** Manpartik S. Gill, Andrew L. Phillips, Danuše Tarkowská, John Addy, Nicolas Virlet, M. John Foulkes, Darren M. Wells, Jonathan A. Atkinson, Stephen Pearce, Stephen G. Thomas, Peter Hedden

**Affiliations:** 1 Rothamsted Research, Harpenden, AL5 2JQ UK; 2 Laboratory of Growth Regulators, Institute of Experimental Botany, Czech Academy of Sciences, Šlechtitelů 27, CZ‑77900 Olomouc, Czech Republic; 3 Laboratory of Growth Regulators, Faculty of Science, Palacký University, Šlechtitelů 27, CZ‑77900 Olomouc, Czech Republic; 4 University of Nottingham, Sutton Bonington, LE12 5RD UK

## Abstract

***Key message*:**

**The wheat** ***dwf4*** **brassinosteroid biosynthesis mutation confers an erect canopy architecture without yield penalties, unlike** ***dwf1*** **, and is a promising target to develop ideotypes suitable for high-density planting.**

**Abstract:**

Brassinosteroids (BR) regulate multiple agronomic traits in plants, including leaf angle. BR depletion promotes an upright canopy architecture, which can improve vertical light distribution and grain yields in cereals under high planting densities. However, broad reductions in BR activity often negatively impact fertility and grain size. Spatially restricted modification of BR metabolism through targeting specific genes could, therefore, provide an erect canopy while minimising negative pleiotropic effects. In the BR biosynthesis pathway, *DWARF1/DIMINUTO* (*DWF1/DIM*) encodes a C-24 reductase that catalyses the production of the first committed sterol BR precursor, while *DWF4* and *DWF11* encode 22α-hydroxylase enzymes that act later in the pathway and exhibit partial functional redundancy. Using ethyl-methanesulphonate (EMS)-induced mutations that introduce premature stop codons, we generated wheat lines lacking functional copies of all three homoeologues of *DWF1* and *DWF4* in the Cadenza background. The *dwf4-abd* mutant displayed upright leaves and a slight height reduction but showed no differences in grain size or weight when tested in glasshouse and field conditions. The *dwf1-abd* mutant also produced an upright canopy, but tiller number, grain size and grain weight were reduced compared to the wild-type. The *dwf1-abd* mutant was hypersensitive to exogenous BR application. Metabolite profiling showed that concentrations of the BR precursors campesterol (CR), campestanol (CN) and 6-oxocampestanol (6-oxoCN) were significantly reduced in *dwf1-abd* seedlings compared to wild-type controls, whereas precursor levels were not reduced in *dwf4-abd* seedlings, consistent with gene redundancy.

**Supplementary Information:**

The online version contains supplementary material available at https://doi.org/10.1007/s00122-026-05418-z.

## Introduction

Brassinosteroids (BRs) are plant hormones that regulate several traits of direct agronomic value, such as stem elongation, leaf angle, and grain size (Nolan et al. 2020; Mantilla-Perez and Salas Fernandez 2017). By promoting cell elongation in the stem, BRs affect overall plant height, and by regulating cell elongation and proliferation in the auricle region of the lamina joint, they influence leaf angle (Sun et al. 2015). Because these traits contribute to crop architecture and yield potential, there is interest in studying natural and induced genetic variation that alters BR biosynthesis and signalling. Characterising this variation can help breed crop varieties with optimal stature, canopy structure and grain production.

Mutations that reduce BR biosynthesis or signalling affect lamina joint development leading to altered leaf angle in cereals such as rice (*Oryza sativa*), maize (*Zea mays*), and barley (*Hordeum vulgare*) (Mantilla-Perez and Salas Fernandez 2017). However, BR-deficient and BR-insensitive plants also exhibit negative pleiotropic effects, such as reduced fertility and grain size, which limit their usefulness for wheat breeding (Morinaka et al. 2006; Dockter et al. 2014; Feng et al. 2016; Gill et al. 2025). By contrast, alleles that confer targeted or partial suppression of BR signalling can lead to enhanced cereal yields. For example, loss of a wheat (*Triticum aestivum* L.) haploblock including *ZnF-B*, which encodes a RING-type E3 ligase that targets a BR-signalling repressor, confers a moderate reduction in BR signalling, resulting in a compact, upright canopy and higher grain yields without negative pleiotropic effects (Song et al. 2023). Similarly, rice lines carrying a mutation that activates the BR catabolic gene *BR-DEFICIENT DWARF3* (*BRD3*) specifically in developing panicles exhibit reduced BR signalling in these tissues, resulting in increased panicle branching and grain number without negative effects on grain size (Zhang et al. 2024).

**Fig. 1:**
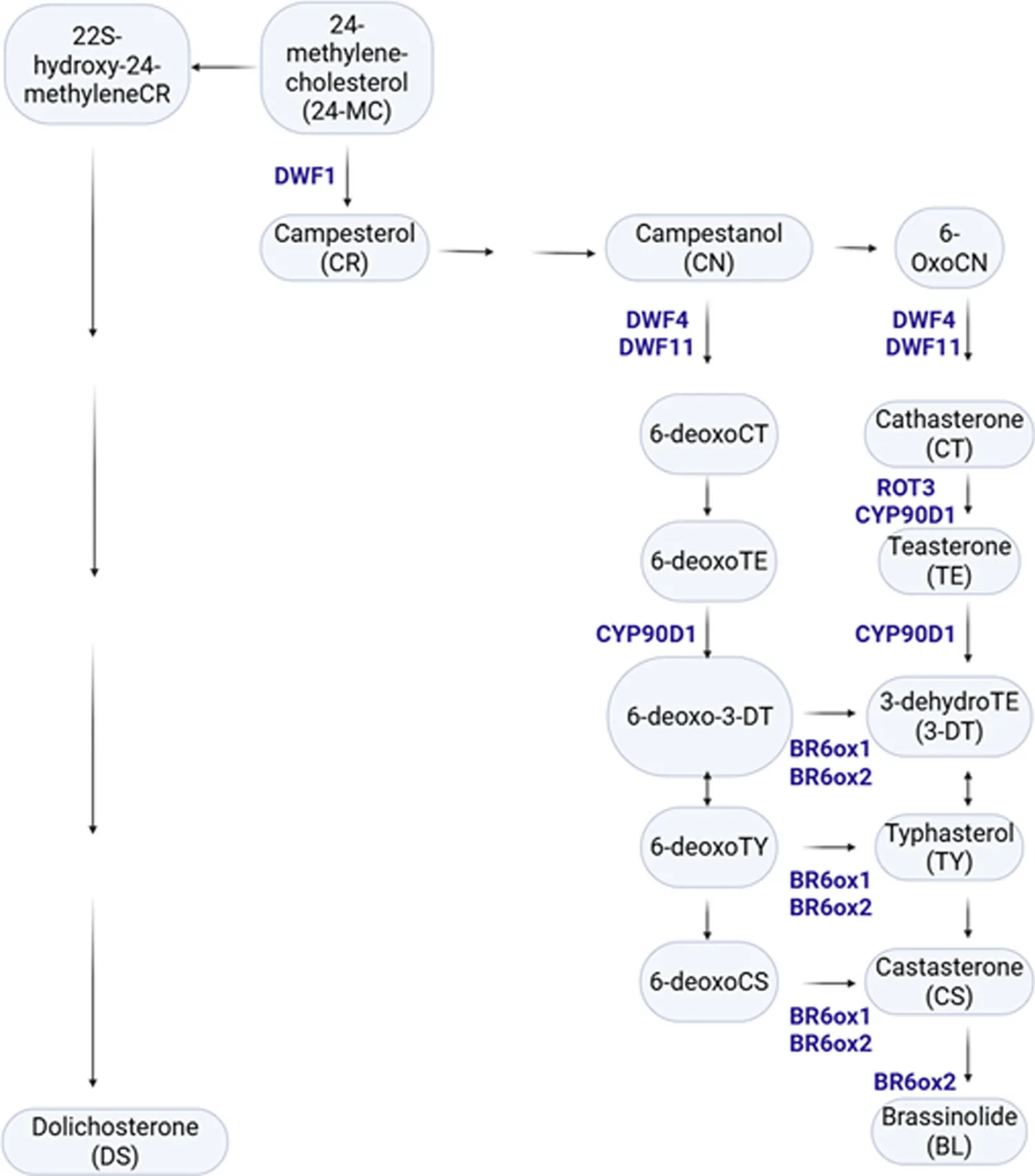
Simplified BR biosynthesis pathway leading to production of BL. The enzymes catalysing each reaction are shown for each step

Another approach to modify BR levels is to target genes encoding BR biosynthesis enzymes. *DWARF1/DIMINUTO* (*DWF1/DIM*) encodes a C-24 reductase that converts 24-methylenecholesterol (24-MC) to campesterol (CR), the committed sterol precursor of BRs, as shown in Fig. 1 (Klahre et al. 1998; Choe et al. 1999). This protein contains an N-terminal transmembrane domain and a flavin adenine dinucleotide-binding (FAD) domain, which is common in oxidoreductase enzymes and essential for its normal function (Choe et al. 1999). Because DWF1 is the only enzyme catalysing this reaction, *dwf1* loss-of-function alleles exhibit severe reductions in BR levels. In Arabidopsis (*Arabidopsis thaliana*), *dwf1* null mutants are BR-deficient and exhibit extremely short hypocotyls, stems, petioles, and roots due to reduced longitudinal cell elongation (Klahre et al. 1998; Takahashi et al. 1995). Loss-of-function mutations in orthologous *DWF1/DIM* genes in barley, rice, and maize also confer erect leaves, semi-dwarf stature, reduced root elongation, shortened grains, and reduced fertility (Dockter et al. 2014; Hong et al. 2005; Best et al. 2016).

By contrast, the 22α-hydroxylation of campestanol (CN) to 6-deoxocathasterone (6-deoxoCT) and of 6-oxoCN to cathasterone (CT), rate-limiting steps in the BR biosynthesis pathway (Sakamoto et al. 2006), are catalysed by two cytochrome P450 monooxygenases in cereals: DWARF4 (DWF4/CYP90B2) and DWF11 (CYP724B1), as shown in Fig. 1 (Sakamoto et al. 2006). Both enzymes contain conserved heme-, dioxygen- and steroid-binding domains that are essential for enzymatic activity (Nebert and Gonzalez 1987; Kalb and Loper 1988). This differs from Arabidopsis, where these steps are catalysed only by DWF4 (Choe et al. 1998).

In rice, *OsDWF4* and *OsDWF11* have partially redundant functions, allowing more targeted reduction in BR activity (Sakamoto et al. 2006). *OsDWF4* transcript levels are highest in leaf blades, and the *osdwf4-1* mutant exhibits an erect-leaf canopy and only a slight reduction in final height with no effects on reproductive development (Sakamoto et al. 2006). By contrast, *OsDWF11* is expressed more broadly, including in vegetative meristems, and the *osdwf11* mutant has a semi-dwarf stature with shorter grains and compact panicles (Sakamoto et al. 2006; Tanabe et al. 2005). The phenotype of the *osdwf4/osdwf11* double mutant is more extreme, including severe dwarfism and malformed leaves (Sakamoto et al. 2006). Therefore, the functional redundancy and distinct expression profiles of *DWF4* and *DWF11* allow the selection of *dwf4* alleles that confer a desirable erect leaf architecture without the negative pleiotropic effects associated with BR-deficient mutants (Sakamoto et al. 2006). This was also demonstrated in maize where loss-of-function *zmdwf4* alleles confer improved photosynthetic capacity, reduced shade avoidance response and higher yields in densely sown plots (Tian et al. 2024).

The wheat genome contains homoeologous triads of *DWF1* on homoeologous group 7 chromosomes, of *DWF11* on homoeologous group 2 chromosomes, and of *DWF4* on homoeologous group 4 chromosomes (Milner et al. 2022). In addition, an inter-chromosomal duplication event specific to wheat originated four *DWF4*-like genes on homoeologous group 3 chromosomes. However, these genes are expressed at very low levels, suggesting they play a minor role in BR biosynthesis (Milner et al. 2022; Gill et al. 2025). To date, genetic variation in BR biosynthesis genes has not been widely exploited in wheat breeding programmes, potentially because the functional redundancy of its hexaploid genome masks the effects of individual gene mutations.

The goal of the current study was to characterise the physiological function of *DWF1* and *DWF4* in bread wheat and determine whether variation in these genes could confer agronomic benefits. Plants carrying loss-of-function mutations in all homoeologous copies of either *DWF1* or *DWF4* exhibited an upright canopy architecture due to anatomical differences in the lamina joint. The *dwf1-abd* mutants were also shorter and produced smaller and lighter grains than the wild-type. By contrast, *dwf4-abd* mutants showed only a slight height reduction and maintained normal grain size, suggesting that *DWF4* is a promising breeding target to develop varieties suitable for dense planting regimes.

## Materials and methods

### Plant materials

The spring bread wheat cultivar Cadenza, supplied by CPB Twyford Ltd, was used in all experiments. Lines carrying ethyl methanesulphonate (EMS)-induced point mutations in *DWF1-A*, *DWF1-B*, *DWF1-D, DWF4-A, DWF4-B* and *DWF4-D* were identified from an online database (http://plants.ensembl.org/Triticum_aestivum/). DNA from M_2_ lines was subjected to exon capture and sequencing to identify mutations (Krasileva et al. 2017). M_3_ seed was grown at the John Innes Centre, which generated and supplied the M_4_ seed. Each M_4_ line was inter-crossed to combine mutations, and F_1_ plants (AaBbDd) were backcrossed twice to non-mutagenized Cadenza to reduce background mutations, then selfed to obtain a BC_2_F_2_ population segregating for each mutation. We selected all single (*tadwf1-a*, *tadwf1-b, tadwf1-d, tadwf4-a, tadwf4-b* and *tadwf4-d*), double (*tadwf1-ab*, *tadwf1-ad*, *tadwf1-bd, tadwf4-ab, tadwf4-ad* and *tadwf4-bd*) and triple (*tadwf1-abd* and *tadwf4-abd*) mutant combinations, as well as the wild-type null segregant line (*TaDWF1-NS* and *TaDWF4-NS*) from this population for phenotypic characterisation.

### Genotyping

Genomic DNA was extracted from seedling tissue using a CTAB-buffer extraction (Aboul-Maaty and Oraby 2019). *DWF1* and *DWF4* genes were amplified to check for the presence of each mutation using 0.05 μM of homoeologue-specific PCR primers (Table S1) with Hot Shot Diamond Mastermix (Clent Life Science, Stourbridge, UK) and annealing temperature between 55–65 ℃ (Table S1). PCR amplicons were purified using QIAquick PCR Purification kit (Qiagen, Hilden, Germany) and sequenced by Eurofins Genomics (Wolverhampton, UK).

*dwf1* and *dwf4* mutations were selected by KASP assays using KASP low-ROX Mastermix (LGC, Teddington, UK) with primers specific to each SNP with FAM, HEX or VIC tails and a common reverse primer with 37 cycles of touchdown PCR (Table S2). Plates were read using the 7500 Fast Software v2.3 (Applied Biosystems, Foster City, California, USA) and analysed using the KlusterCaller™ software (LGC, Teddington, UK).

### Phenotypic characterisation of *dwf1 *and* dwf4* mutants under glasshouse conditions

Preliminary phenotyping of all *dwf1* and *dwf4* combinatorial mutants was performed under glasshouse conditions (18–20℃ day/14–15℃ night temperatures, 16 h photoperiod) using four biological replicates, organized in a randomized complete block design. More detailed phenotyping of *dwf1-abd* and *dwf4-abd* mutants used six biological replicates, also sown in a randomised complete block design. Wheat grains were imbibed on damp filter paper at 4 ℃ for 4–5 d, then transferred to 15 cm pots containing 75% peat, 12% sterilised loam, 3% vermiculite and 10% grit.

Leaf angle was defined as the angle between the vertical stem and the midrib. Measurements were made using a protractor for the 2^nd^ leaf at the seedling stage (GS12) and the flag leaf at anthesis (GS61) and at 14 days post-anthesis (DPA) (GS73) on a representative primary shoot. Leaf width and length were measured on the 2^nd^ leaf at the widest and longest point of the leaf. The leaf area was calculated using the equation: length x width x 0.835 (Miralles et al. 1998). Final plant height was measured from the soil surface to the tip of the spike of the tallest shoot at maturity (GS97). Internode and spike lengths (from the base of the first spikelet to the top of the terminal spikelet, just beneath the awns) were measured from the 3 tallest shoots per plant. The number of fertile spikelets per spike was measured from 2–3 spikes per plant. Grain area was determined using a Marvin grain analyser (INDOSAW, India) with 3–5 g of clean threshed grain. Thousand Grain Weight (TGW) was measured by weighing 1,000 clean threshed grains.

### Field assessment of *dwf1 *and* dwf4* mutants

The genotypes *dwf1-abd, dwf4-abd* and Cadenza were sown in April 2022 at Rothamsted Research Experimental farm in 1 m^2^ plots as a randomized complete block design with four replicates. All experiments were grown with standard farm practice for fertiliser and pesticides, but no plant growth regulators were applied. Final plant height, spike length, number of spikelets per spike, grain area, and TGW were measured as in the glasshouse experiment using 10–15 plants per plot. Flag leaf angle (FLA) was measured using a protractor on 10–15 uniform tillers at anthesis. Final plant height was recorded as the mean of the 10 tallest fertile shoots per plot.

### High-throughput phenotyping under the Field Scanalyzer

For seed multiplication, *dwf1-abd* and Cadenza were sown in 1 m^2^ plots in Rothamsted Research Experimental Farm in spring 2019. The row-to-row spacing was 20 cm, and plant-to-plant spacing was 10 cm, with a 60 plants/ m^2^ plot density. These genotypes were sown in autumn 2020 under the Field Scanalyzer, located in Rothamsted Research Experimental Farm. Three biological replicates of both genotypes were grown in 0.6 m^2^ plots as a randomised complete block design. Data were captured using the red, green and blue (RGB) camera and the 3D laser scanners from seedling establishment to harvesting (Virlet et al. 2016). RGB images were used to calculate the canopy cover (ratio of green pixels to the total amount of pixels within the image) (Sadeghi-Tehran et al. 2017) and to quantify the number of spikes per image, then converted to spike density (spikes/m^2^) (Sadeghi-Tehran et al. 2019). The 3D point clouds collected by the 3D laser scanners were processed to measure the canopy height using methods described previously (Lyra et al. 2020).

### Brassinosteroid analysis

Bioactive and precursor BR concentrations were determined in 2^nd^ leaf seedling tissues of *dwf1-abd, DWF1-NS, dwf4-abd, DWF4-NS*, and Cadenza. Multiple plants were pooled to obtain sufficient tissue for each of four biological replicates. Leaf tissue was flash-frozen in liquid N_2_ and freeze-dried. BRs were extracted using 5 mg DW of tissue, isolated by one-step solid-phase extraction, and analysed by UHPLC-ESI–MS/MS (Acquity UPLC™ I-Class PLUS system coupled to a triple-stage quadrupole mass spectrometer Xevo® TQ XS; both Waters, USA) as described previously (Tarkowská et al. 2016).

### Lamina joint inclination assay

Two-cm sections containing the 2^nd^ leaf lamina joint, leaf sheath, and leaf blade were excised from uniformly developed seedlings and floated in autoclaved water for 10 min in Petri dishes. These sections were then transferred to Petri dishes containing either 10^−5^ M *epi*BL (24-*epi*brassinolide) solution or autoclaved water. The dishes were sealed with parafilm and incubated at 29 °C in the dark for 2 d. Leaf angles were subsequently measured using a protractor (Li et al. 2017).

### Scanning electron microscopy (SEM)

Seedlings were grown in the glasshouse for 10–14 d under a 16 h photoperiod. Leaf segments (~ 1 cm long), including the second leaf lamina joints, were harvested and attached to aluminium stubs using a 50:50 mixture of graphite and TissueTek. Samples were immediately immersed in liquid nitrogen, then transferred to a GATAN ALTO 2100 cryo-prep system, sputtered with argon and coated with a thin layer of gold. Micrographs were captured using a JEOL JSM-6360LV scanning electron microscope, and cell lengths were measured using ImageJ 1.48v software (Schneider et al. 2012).

### Laser ablation tomography (LAT)

Wheat seedlings were grown for 11–12 d under 16 h photoperiod (21 °C day/16 °C night temperature) in a growth chamber. Leaf segments (approx. 1 cm) including the lamina joints of the 2^nd^ leaf were harvested and dipped in fresh water for 5–10 min. These leaf segments were transferred to 100% methanol (fixation) for 2 d at 4 ºC and then transferred to 100% ethanol (dehydration) for 2 d at 4 ºC. The samples were then transferred to a critical point dryer (Leica EM CPD300) to remove the liquid without affecting the cellular structure. Dried samples were stored in sealed containers with silica gel desiccant until analysis, when they were attached to a magnetic clamp and transferred to the LAT system (LatScan 2.0, L4IS). The lamina joint was imaged in full from base to tip, with images captured at 10 μm intervals at 5X magnification. As consecutive cross-sections were highly similar, images were sampled at 100 µm intervals, yielding approximately five to six representative cross-sections across the ~400–500 µm region. Imaging was performed on 10 biological replicates per genotype. For each cross-section, the width and number of cell layers adjacent to the abaxial (D1) and adaxial (D2) regions, the lamina-joint width (C), and the mid-vein-to-adaxial distance (D3) were measured in ImageJ. The number of cell layers in D1 and D2 were counted manually. Average cell-layer thickness was subsequently derived as region length divided by the corresponding cell-layer count. The detailed procedure for critical point drying, LAT and image capture is described in Jones (2023).

### Statistical analysis

Analysis of variance (ANOVA) was conducted on the glasshouse data using Genstat software (Release 21.1) to estimate statistical differences between genotypes and treatments, from which *p-*values, standard errors of the difference of the means (SED), and least significant differences (LSD) at the 5% level were calculated. Field data were analysed using unbalanced ANOVA with regression. Differences between genotypes were further tested using Fisher’s LSD unprotected test, providing *p-*values. Paired Student’s *t*-tests, which provided *p*-values, were performed in GraphPad Prism software (version 9.4.0) to compare means in phenotyping data from the Field Scanalyzer. Two-way ANOVA and F-test were performed in R (R version 4.4.1) to test the sensitivity and interaction of genotypes to *epi*BL treatment, respectively. The significance level was set to 0.05. Mean and residual plots were generated for each dataset to assess normality. For the cell and organ length measurements provided by SEM and LAT microscopy, one-way ANOVA was performed to estimate statistical significance, followed by Fisher’s LSD test for significant differences amongst the genotypes. For the number of cell layers in D1, D2, a quasi-Poisson Generalized Linear Model was used to fit the data, followed by an F-test and ANOVA to test significance and Fisher’s LSD to test differences between these means.Graphs were made with GraphPad Prism (version 9.4.0) and R software (R version 4.4.1).

## Results

### Generation of *dwf1* and *dwf4* mutants

We generated *dwf1* and *dwf4* knockout mutants in wheat by identifying EMS-induced point mutations that introduce premature stop codons in each of the three respective homoeologues (Table 1, Fig. S1). The *dwf1* mutant alleles each encode truncated proteins lacking either the FAD or transmembrane domains (Fig. S1A, B). Similarly, the *dwf4* mutant alleles encode proteins lacking conserved domains, including the heme-binding domain, that are required for function (Fig. S1C, D). Heterozygous triple mutants were back-crossed twice with Cadenza to reduce the number of background EMS-induced mutations before selfing. However, residual non-target mutations may still have contributed to the observed phenotypes. We selected single, double, and triple mutant lines for both *DWF1* and *DWF4*, as well as their wild-type null segregants, for phenotypic characterisation.

**Table 1:** *DWF1* and *DWF4* homoeologues and mutant alleles used in this study

Gene	Gene ID	Mutant name	Amino acid substitution	Cadenza TILLING line
*DWF1-A*	*TraesCS7A02G559400*	*dwf1-a*	W92*	381
*DWF1-B*	*TraesCS7B02G484200*	*dwf1-b*	Q23*	1444
*DWF1-D*	*TraesCS7D02G550700*	*dwf1-d*	Q252*	1339
*DWF4-A*	*TraesCS4A02G078000*	*dwf4-a*	W168*	1353
*DWF4-B*	*TraesCS4B02G234100*	*dwf4-b*	W39*	12
*DWF4-D*	*TraesCS4D02G235200*	*dwf4-d*	W352*	934

The position of mutations introducing premature stop codons (*) are indicated

### Phenotypic characterization of *dwf1* and *dwf4* mutants under glasshouse conditions

To characterize *DWF1* and *DWF4* function in wheat, we measured plant height and FLA in single, double, and triple knockout mutants. We observed mild but significant reductions in FLA in all single and double knockout *dwf1* mutants compared to *DWF1-NS*, except *dwf1-ab* (Fig. S2A). The reduction in FLA was much greater in the triple *dwf1-abd* mutant (Fig. S2A). Height and internode lengths were unaffected in any single or double *dwf1* mutant, but were significantly reduced in the *dwf1-abd* mutant (Fig. S2B and D). Tiller number was reduced in *dwf1-abd*, but not in the single and double *dwf1* mutants (Fig. S2C). All single and double *dwf4* mutations also exhibited reduced FLA compared to *DWF4-NS,* although the difference was significant only for *dwf4-bd* (Fig. S3A). As for *dwf1-abd*, FLA was significantly reduced in the *dwf4-abd* triple mutant.While plant height and internode lengths were not significantly affected in any single, double or triple *dwf4* mutant (Fig. S3B and C), there was a slight reduction in spike length in *dwf4-ad* and *dwf4-abd* (Fig. S3E). Tiller number was slightly reduced in *dwf4-abd* and the *dwf4-ad* and *-bd* double mutants but not in any other *dwf4* mutant (Fig. S3D).

**Fig. 2:**
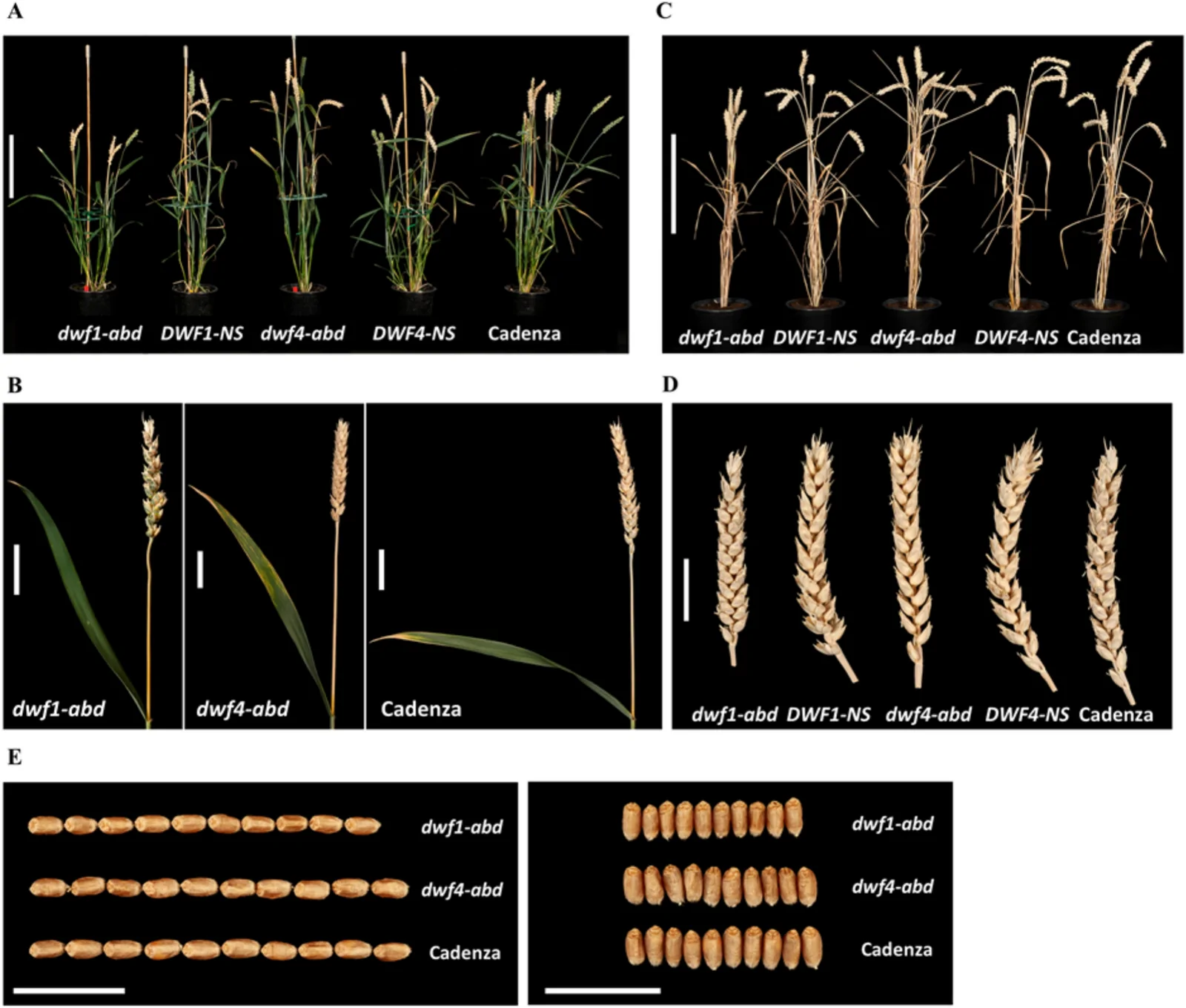
Phenotype of *dwf1* and *dwf4* triple mutants. (**A**) *dwf1-abd*, *dwf4-abd,* respective null segregant lines and Cadenza photographed at the hard dough stage (GS87) (Bar length = 20 cm). (**B**) Flag leaf and main stem indicating leaf angles at GS87 (Bar length = 3 cm). (**C**) Mature plants and (**D**) spikes of *dwf1-abd*, *dwf4-abd*, *DWF1-NS*, *DWF4-NS* and Cadenza at GS97. Bar lengths are 30 cm and 3 cm, respectively. (**E**) Ten grains end-to-end and side-by-side of *dwf1-abd*, *dwf4-abd* and Cadenza at maturity (Bar length = 2 cm)

**Fig. 3:**
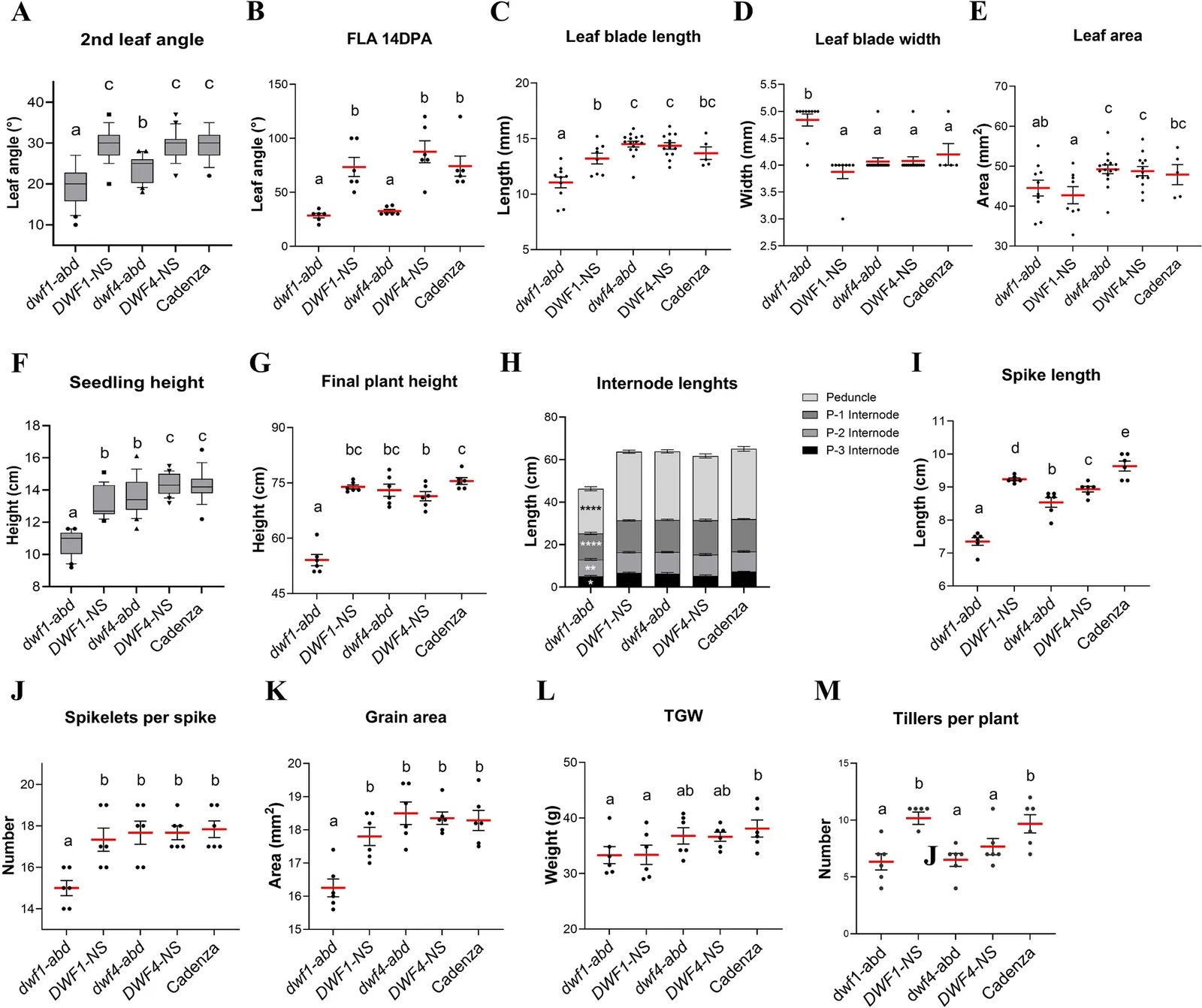
Phenotypic characterization of *dwf1* and *dwf4* mutants under glasshouse conditions. (**A**) Leaf angle at 2^nd^ leaf seedling stage, GS11 (n ~ 20). (**B**) Flag leaf angles (FLA) at 14 days post-anthesis, GS73 (n = 6). (**C** and **D**) Leaf blade length and width at 2^nd^ leaf seedling stage (n=10). (**E**) Leaf area of the 2^nd^ leaf at the seedling stage (n=10). **F** Height at 2^nd^ leaf stage (n ~ 20). (**G**) Final plant height and (**H**) internode lengths of mature stems (n = 6). (**I**) Spike length at maturity (n = 6). (**J**) Number of spikelets per spike (n = 6). (**K**) Area and (**L**) TGW of mature grains (n = 6). (**M**) Number of tillers per plant (n = 6). Different letters indicate significant differences between genotypes (obtained from Fisher’s unprotected LSD test). *p* values *0.05>*p*>0.01, **0.01 > *p *> 0.001, ****0.0001 > *p *> 0.00001 (obtained from Fisher’s unprotected LSD test)

Because of the major reduction in FLA in *dwf1-abd* and *dwf4-abd* mutants, we performed a more detailed phenotypic characterization of these lines, along with their respective null segregant lines (*DWF1-NS* and *DWF4-NS*) and Cadenza. The gross morphology of these lines at the hard dough stage (GS87) and maturity (GS97) is shown in Fig. 2A and C, respectively. Leaf angle was significantly reduced at the 2^nd^ leaf and flag leaf stages in both *dwf1-abd* and *dwf4-abd* mutants compared with their respective null segregant controls (Figs. 2A, B and 3A, and B). The reduction was greater in *dwf1-abd* than in *dwf4-abd* at the 2^nd^ leaf seedling stage, while at the flag leaf stage, the leaf angles in these mutants were similar and significantly reduced relative to their respective null segregants. This result suggests that similar regulatory mechanisms control leaf angle at both the seedling and reproductive stages. The 2^nd^ leaf blade was shorter, and wider in the *dwf1-abd* mutant compared to the *DWF1-NS* (Fig. 3C and D). The leaf blade areas of the *dwf1-abd* and *dwf4-abd* mutants were not significantly different from that of Cadenza or the respective null segregants, but the leaf area of *dwf1-abd* was significantly smaller than that of *dwf4-abd* (*p* < 0.05, Fig. 3E).

Both *dwf1-abd* and *dwf4-abd* seedlings were significantly shorter than their respective null segregant controls (Fig. 3F). However, only *dwf1-abd* showed a significant reduction in final plant height (Figs. 2C and 3G), due to decreases in spike length and the upper four internodes (Fig. 3H and I). In addition, *dwf1-abd* produced fewer spikelets per spike, smaller and lighter grains, and fewer tillers per plant compared to *DWF1-NS*, whereas *dwf4-abd* did not significantly differ from *DWF4-NS* for these traits (Figs. 2D, E and 3J-M).

### *dwf1* and *dwf4* mutants display anatomical differences in the lamina joint

**Fig. 4:**
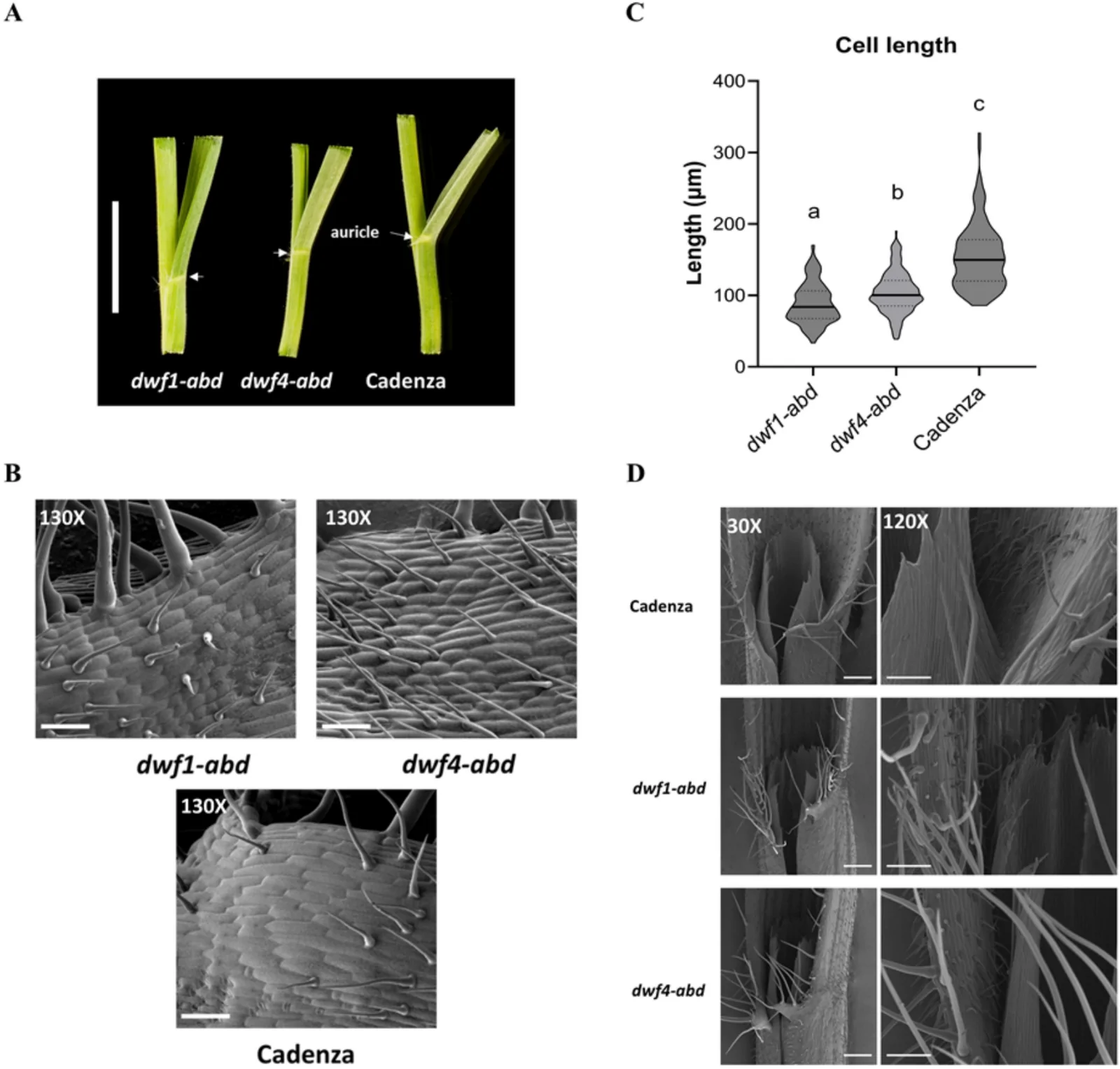
Scanning electron microscopy imaging around the lamina joint in the *dwf1* and *dwf4* mutants and Cadenza. (**A**) The leaf segments containing the lamina joint of the 2^nd^ leaf in *dwf1-abd*, *dwf4-abd,* and Cadenza (Bar length = 1 cm). The auricle region is smaller in both mutants compared to Cadenza, as shown by arrows. (**B**) Images taken around the auricle region of the lamina joint (Magnification = 130X, scale bar = 100 µm). (**C**) Cell length from images of *dwf1-abd* (n = 197), *dwf4-abd* (n = 200) and Cadenza (n = 131). Different letters indicate significant differences between the genotypes obtained from Fisher’s LSD unprotected test. (**D**) Imaging around the adaxial side of the lamina joint. (Left image, magnification = 30X, scale bar = 500 µm; right image magnification = 120X, scale bar = 200 µm)

**Fig. 5:**
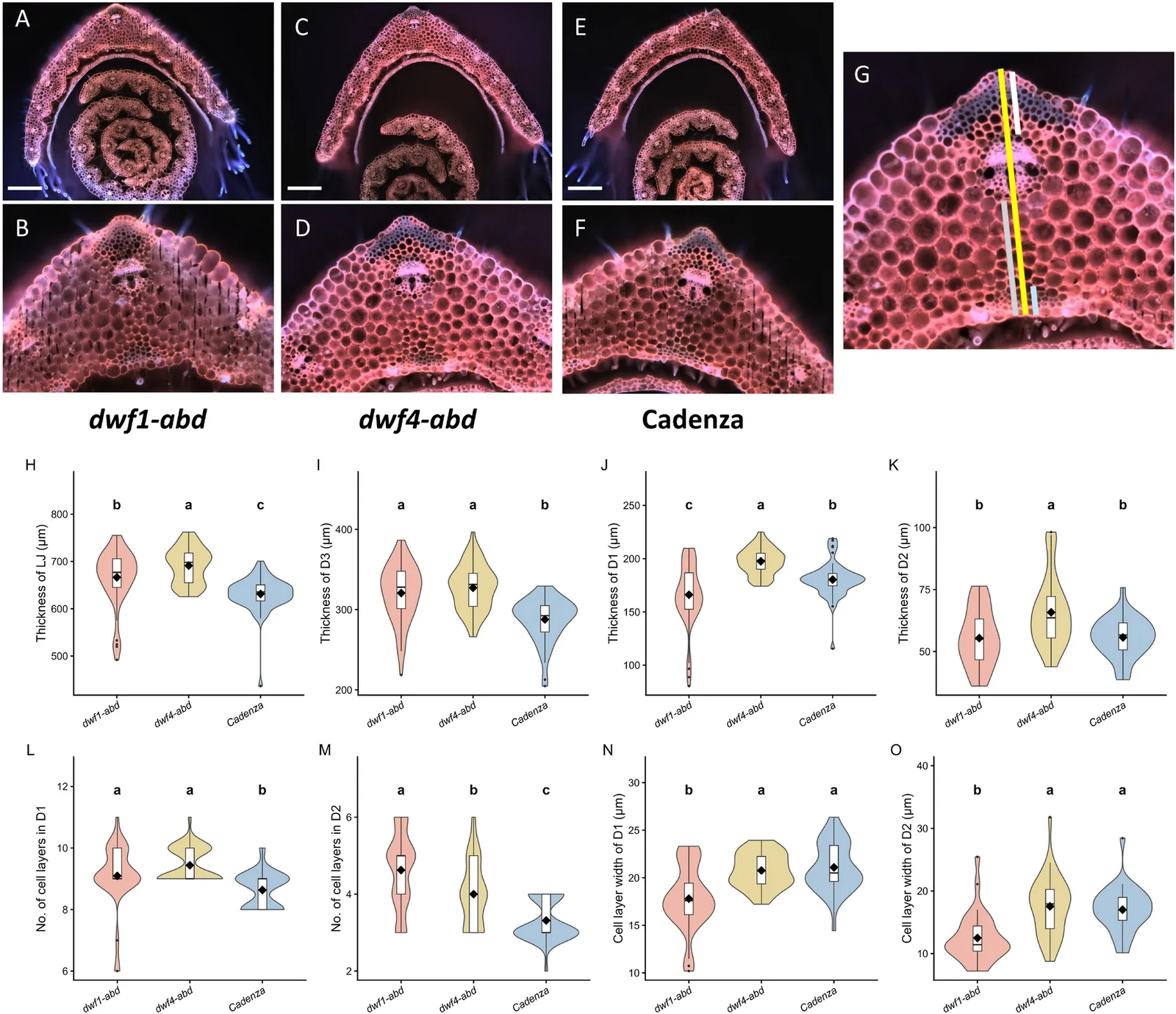
Laser ablation tomography (LAT) imaging in the lamina joint. LAT images were captured on the lamina joint of *dwf1-abd* (**A** and **B**), *dwf4-abd* (**C** and **D**) and Cadenza (**E** and **F**) at the 2^nd^ leaf seedling stage. The scale bar length is 500 *μ* m in (**A**, **C** and **E**) and images were captured at 5X magnification. (**B**, **D** and **F**) show these images in close-up. (**G**) shows different regions of the lamina joint measured, C (yellow) is the thickness of the entire lamina joint, D3 (grey) is the distance from the mid vein to the adaxial end, D1 (white) and D2 (blue) are the thickness of the indicated regions adjacent to the abaxial and adaxial surfaces, respectively. (**H**) shows the thickness of the entire lamina joint (n = 55–60), (**I**, **J**, and **K**) show the thickness of D3 (n = 55–60), D1 (n = 55–60) and D2 (n = 30–35), respectively. (**L** and **M**) show the number of cell layers in the D1 (n = 30–35) and D2 (n = 30–35) regions, respectively. (**N** and **O**) show the average cell layer widths in the D1 (n = 30–35) and D2 (n = 30–35) regions. Different letters indicate significant differences between genotypes (obtained from Fisher’s unprotected LSD test)

We used SEM and LAT to investigate anatomical differences around the auricle (Fig. 4A) and adaxial lamina joint regions of the 2^nd^ leaf. Both *dwf1-abd* and *dwf4-abd* exhibited significantly shorter average cell lengths (*p* < 0.0001) in the auricle compared to Cadenza (Fig. 4B and C). In addition, the leaf blade was more tightly packed around the ligule in both mutants than in Cadenza (Fig. 4D). The lamina joint was significantly thicker (*p* < 0.001) in *dwf1-abd* and *dwf4-abd* mutants compared to Cadenza (Fig. 5A-F and H), which was due to a greater distance between the mid vein and the adaxial border (D3 in Fig. 5G and I). The abaxial and adaxial cell layers (D1 and D2) were significantly wider in *dwf4-abd* compared to *dwf1-abd* and Cadenza (Fig. 5J and K). Additionally, there were more cell layers in D1 and D2 in both mutants than in Cadenza (Fig. 5L and M), with significantly narrower cell layers in *dwf1-abd* mutant compared to other genotypes (Fig. 5N and O).

### Response of *dwf1* and *dwf4* mutants to BR application

**Fig. 6:**
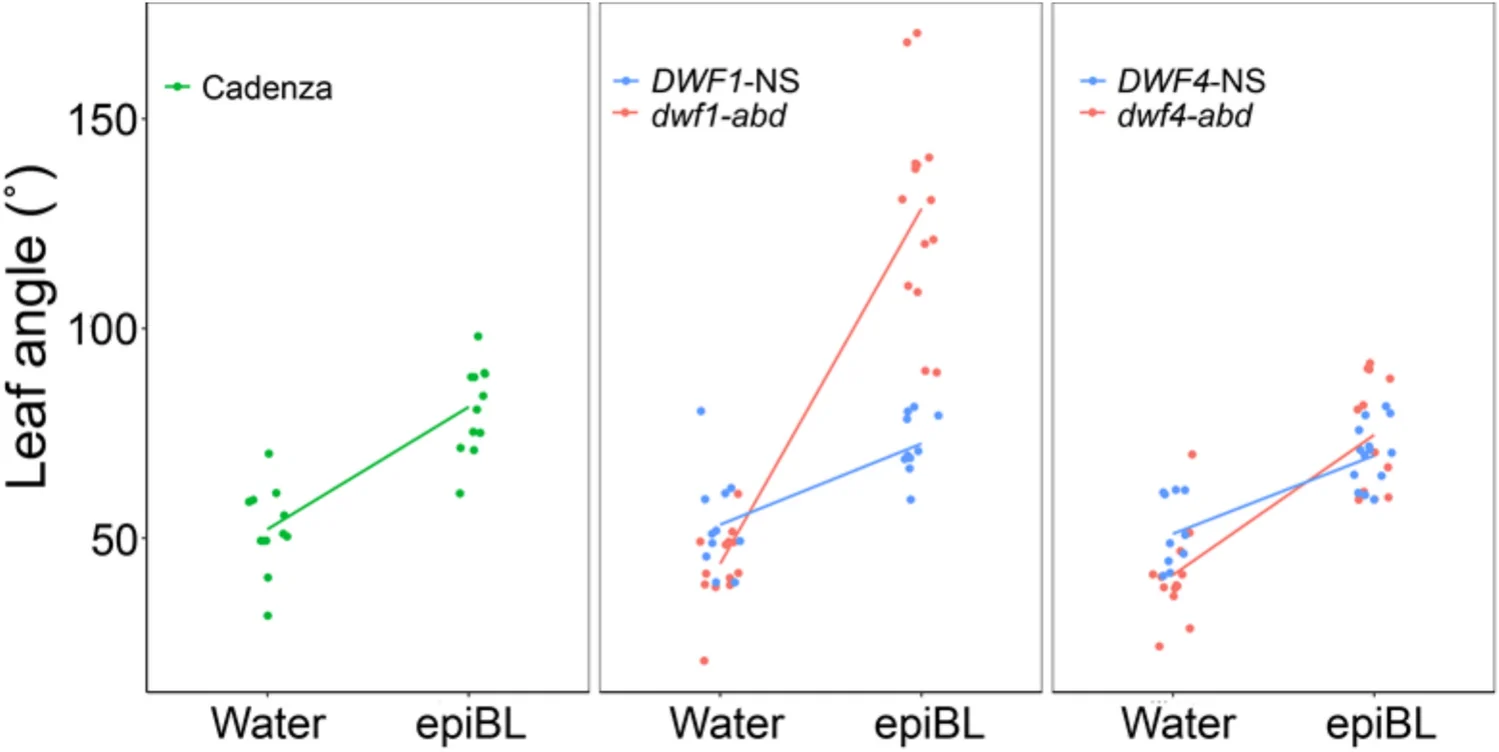
Response of *dwf1* and *dwf4* mutants to external BR application. Lamina joint inclination assays were performed on *dwf1-abd*, *DWF1-NS*, *dwf4-abd, DWF4-NS* and Cadenza. Leaf angles of the 2nd leaf at the seedling stage (n = 14) were measured after treatment with 10^–5^ M *epi*BL or water. The data were analysed using two-way ANOVA, where an F-test was used to test for the interaction

To test the sensitivity of *dwf1-abd* and *dwf4-abd* mutants to exogenous BRs, we applied *epi*BL to seedling sections of the mutants, their respective null segregants and Cadenza and measured leaf angles in lamina joint inclination assays. While all lines responded to the treatment with an increase in leaf angle, there was a significant interaction between treatment and genotype, with *dwf1-abd* responding much more strongly to 10^−5^ M *epi*BL compared to the other lines (F_4,111_ = 28.892, *p* <0.001) (Fig. 6).

### Analysis of endogenous BR levels in *dwf1 *and* dwf4* triple mutants

**Fig. 7:**
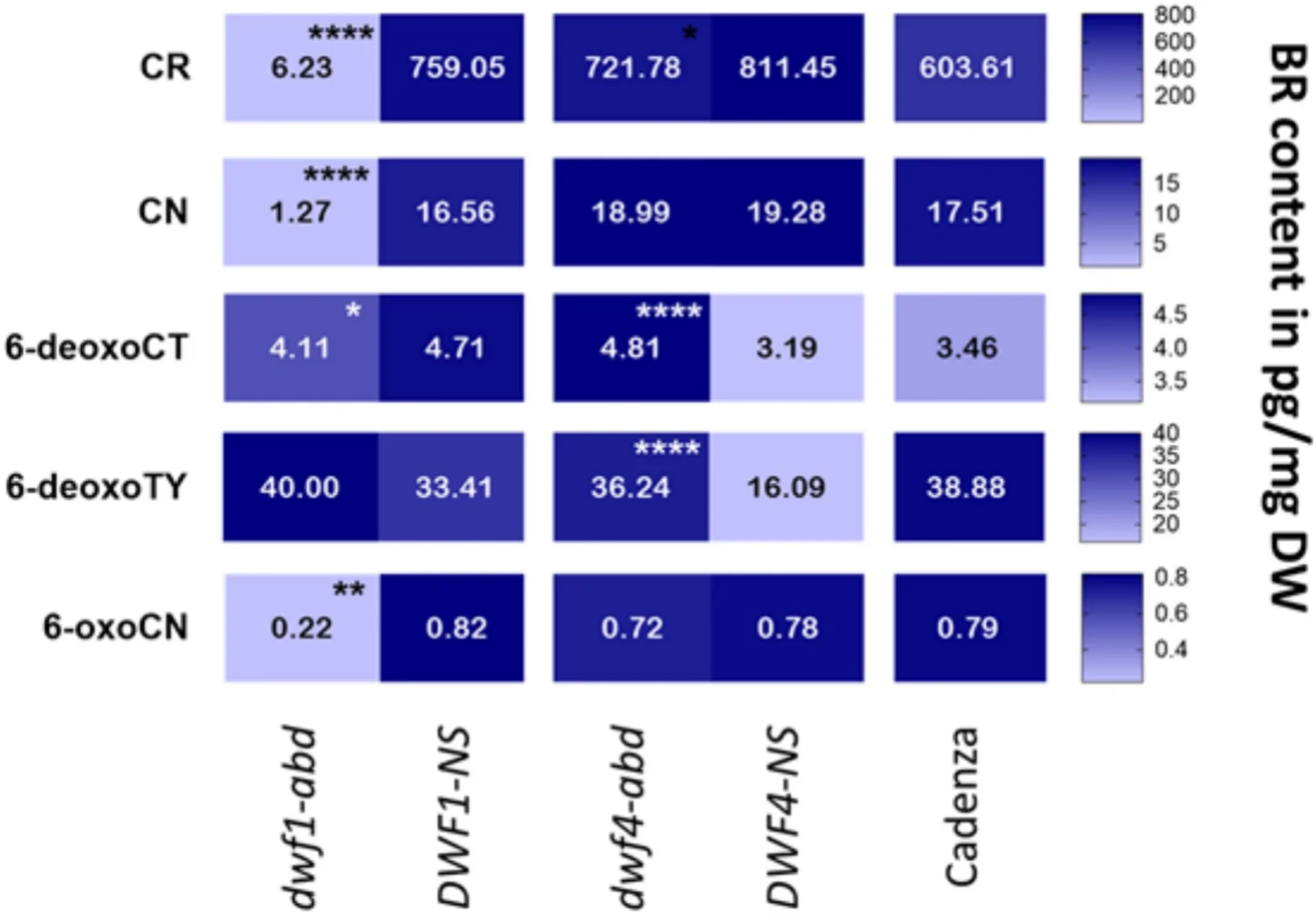
BR levels in *dwf1* and *dwf4* mutants*.* The concentrations of BRs (pg/mg DW), namely CR (campesterol), CN (campestanol), 6-oxoCN, 6-deoxoCT (6-deoxocathasterone), and 6-deoxoTY, were determined in seedlings of the *dwf1-abd, dwf4-abd* mutants, *DWF1-NS*, *DWF4-NS* and Cadenza. Mean values from five biological replicates are recorded. Statistically significant differences from the respective *NS* control are denoted by * 0.05 > *p* > 0.01, ** 0.01 > *p* > 0.001, **** *p* < 0.0001 (obtained by pairwise Student’s *t*-test)

We analysed nine BRs in seedling tissues of Cadenza, *dwf1-abd*, *dwf4-abd* and their null segregants (Gill et al. 2026). In *dwf1-abd*, the early sterol intermediates CR, CN, and 6-oxoCN levels were reduced by 126-fold, 12.8-fold, and 3.7-fold, respectively, compared to *DWF1-NS* (Fig. 7, Table S3). The concentrations of the bioactive BRs castasterone (CS) and brassinolide (BL) as well as those of typhasterol (TY), and dolichosterone (DS) were extremely low, below the limit of detection, and we could not obtain reliable values for them. The concentration of CN, the substrate for DWF4 and DWF11, did not differ significantly between *dwf4-abd* and *DWF4-NS* (Fig. 7). Moreover, 6-deoxoCT, the immediate product of these enzymes, as well as 6-deoxytyphasterol (6-deoxoTY), accumulated to significantly higher levels in *dwf4-abd* than in *DWF4-NS.* However, the concentrations of both compounds were significantly lower in *DWF4-NS* than in *DWF1-NS* or Cadenza.

### High-throughput phenotyping of the *dwf1* mutant using a Field Scanalyzer

**Fig. 8:**
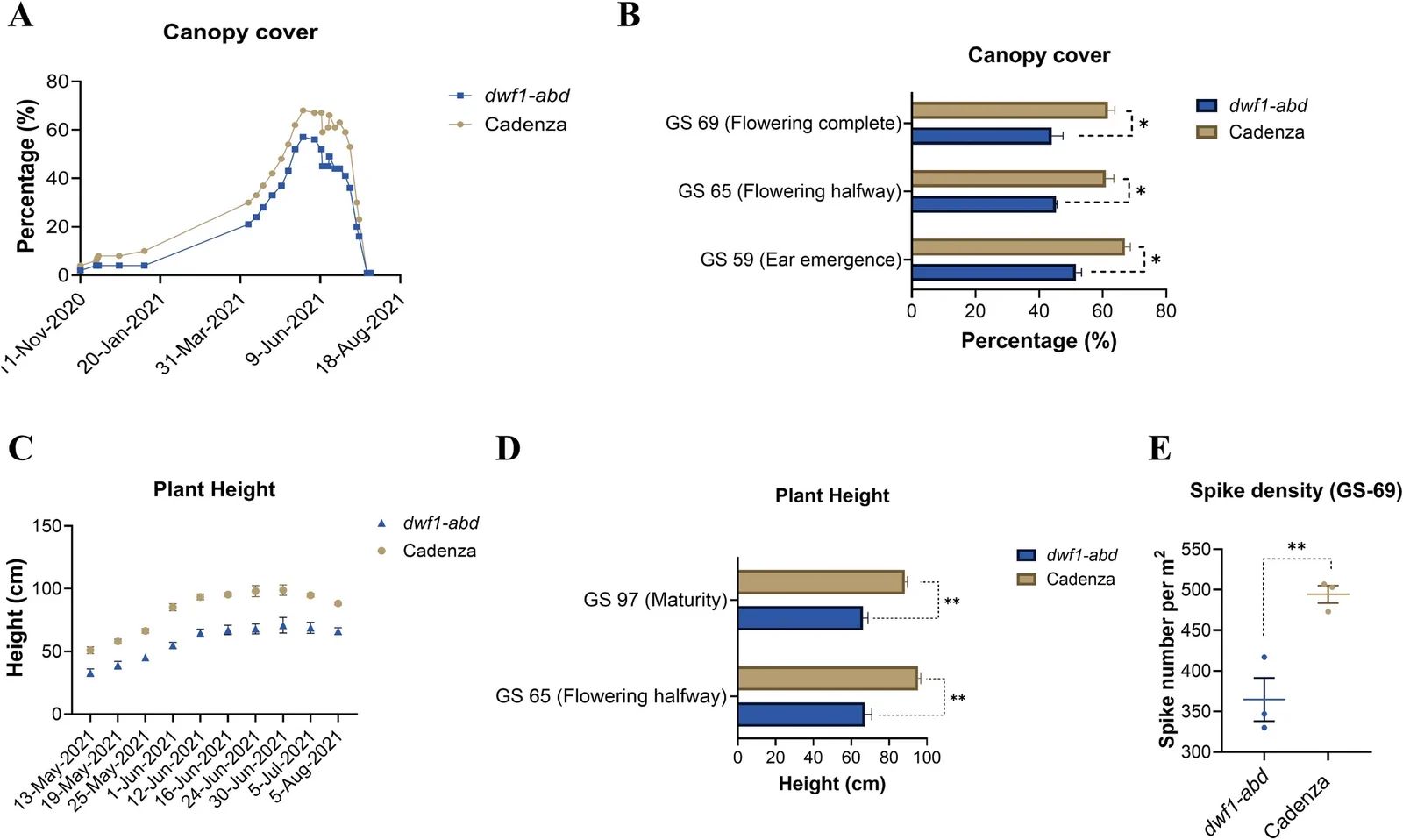
High-throughput phenotyping of *dwf1* mutant under a Field Scanalyzer. (**A**) Canopy cover was estimated from these RGB images throughout the life cycle of the crop (**B**) and specifically at flowering stages GS59 (spike emergence), GS65 (flowering halfway), and GS69 (flowering complete) (n = 3). Significant differences were denoted as * *p* < 0.05 from the one-tailed Student’s *t*-test. (**C**) Plant height was recorded in *dwf1-abd* and Cadenza from mid-May 2021 (initiation of internode elongation) to August 2021 (maturity). (**D**) Heights of these genotypes at the GS65 and GS97 (maturity) **p < 0.01 (obtained from one-tailed Student’s *t*-test). (**E**) RGB images were used to estimate spike density (number of spikes per m^2^) in *dwf1-abd* and Cadenza (n = 3). **p < 0.01 (obtained from one-tailed Student’s *t*-test)

When multiplying *dwf1-abd* seed in field plots in 2019, we noted obvious reductions in height and canopy cover compared to Cadenza (Fig. S4). To investigate canopy cover, spike density, and stem elongation patterns throughout the growing season, we grew *dwf1-abd* alongside Cadenza under the Field Scanalyzer in winter 2020. From early November 2020 to late July 2021, *dwf1-abd* consistently exhibited lower canopy cover than Cadenza, including during flowering (Figs. 8A, B and S5). The *dwf1-abd* plants were shorter than Cadenza throughout the reproductive stage (Fig. 8C), with height reductions of ~30% at GS65 and ~25% at GS91 (Fig. 8D). In addition, spike density at GS69 was significantly lower in *dwf1-abd* compared to Cadenza (Figs. 8E and S5).

### Phenotypic characterization of *dwf1* and *dwf4* mutants under field conditions

**Fig. 9:**
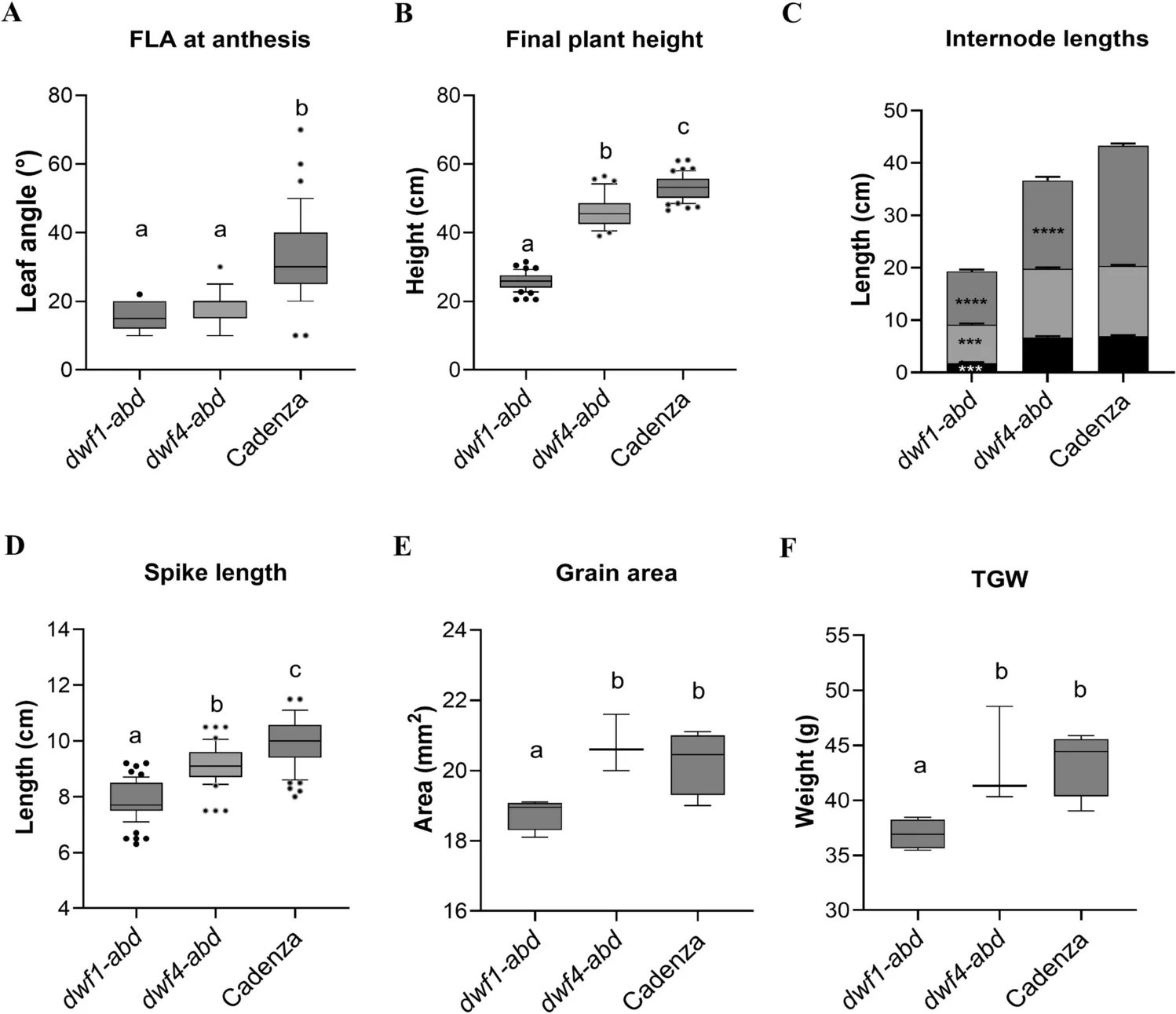
Phenotypic characterization of *dwf1-abd* and *dwf4-abd* mutants in the field during 2022. (**A**) Flag leaf angle at anthesis (n ~ 40). (**B**) Final plant height, (**C**) Internode lengths and (**D**) spike lengths at maturity (n = 55–60). (**E**) Grain area and (**F**) TGW of mature grains (n = 4). Different letters indicate significant differences amongst the genotypes (obtained from Fisher’s unprotected LSD test). *p* values ***0.001> *p *> 0.0001, ****0.0001 > *p *> 0.00001 (obtained from Fisher’s unprotected LSD test)

We compared the *dwf1-abd* and *dwf4-abd* mutants with Cadenza in a full, replicated field experiment sown in spring 2022. Both mutants exhibited significantly reduced FLAs compared to Cadenza (Fig. 9A). The *dwf1-abd* mutant was ~50% shorter than Cadenza due to significant reductions in the lengths of the spike, peduncle, P-1 and P-2 internodes (Fig. 9B-D). The reduction in height in *dwf1-abd* was approximately double the difference observed in the earlier winter-sown experiment (Fig. 8C and D), suggesting that environmental conditions impact the expression of these traits. Grain area and TGW were also significantly lower in *dwf1-abd* compared to Cadenza (Fig. 9E and F).

The *dwf4-abd* mutant was also significantly shorter than Cadenza (14% reduction), a milder effect than in the *dwf1-abd* mutant (Fig. 9B), and only the peduncle and spike were significantly shorter (Fig. 9C and D). Grain size and TGW did not differ from Cadenza (Fig. 9E and F).

Taken together, the *dwf1-abd* mutant displayed compact architecture with smaller, lighter grains, whereas the *dwf4-abd* mutant exhibited an erect leaf canopy and a mild height reduction without any detrimental changes in grain characteristics.

## Discussion

### *DWF1* is required for normal above-ground architecture in wheat

The *dwf1-abd* triple mutant displayed striking developmental defects under both glasshouse and field conditions, including a ~50% reduction in height due to restricted internode elongation, erect, shorter but wider leaves, and a compact spike. Grain size and weight were also significantly lower than in the wild-type. Similar phenotypes have been reported for *DWF1* knockouts in rice, barley, and maize, demonstrating a conserved role in promoting stem elongation and grain filling (Dockter et al. 2014; Hong et al. 2005; Best et al. 2016).

High-throughput canopy imaging using Rothamsted’s Field Scanalyzer revealed that green canopy cover was significantly reduced in *dwf1-abd* plots compared to Cadenza throughout the growing season (Fig. 8). While it is not possible to exclude the effect of different germination rates, which was not recorded in this experiment, the lower canopy cover for the *dwf1-abd* plots is likely due to its more erect phenotype (Hong et al. 2005; Best et al. 2016). This is also supported by the lower spike density at GS69 of *dwf1-abd* plots compared to Cadenza, indicating a lower number of fertile tillers contributing to lower canopy cover. This is consistent with studies in rice showing that BR signalling stimulates axillary bud outgrowth, promoting tillering (Fang et al. 2020). Although it was not measured in the present study, reduced tiller angle could also contribute to the attenuated canopy cover (Zhang et al. 2014), as shown in Fig. S4. Taken together, the combination of reduced height, lower tillering and smaller grains means that *dwf1* mutations are unlikely to be beneficial in wheat breeding.

The *dwf1-abd* mutant was hypersensitive to exogenous BL, and the concentrations of CR, the immediate product of DWF1, were 126-fold lower than in wild-type control plants, consistent with BR-deficiency (Klahre et al. 1998; Youn et al. 2018) (Fig. 7). The concentrations of downstream intermediates were also reduced in this mutant, consistent with results from *dwf1* mutants in rice and Arabidopsis (Klahre et al. 1998; Choe et al. 1999; Hong et al. 2005), although the later intermediate 6-deoxoTY was not significantly reduced in wheat *dwf1-abd* mutants compared to either null segregant or Cadenza control lines. It is noteworthy that the difference in BR concentrations between *dwf1-abd* and its null segregant decreased with progression through the biosynthetic pathway, consistent with compensatory homeostatic mechanisms involving up-regulation of multiple biosynthesis genes (Tanaka et al. 2005). Nevertheless, *dwf1-abd* has a clear phenotype characteristic of BR deficiency, suggesting spatial specificity in BR distribution. Unfortunately, bioactive BRs, CS and BL could not be reliably quantified because they were below the limit of detection. It is unclear how CR and subsequent intermediates are produced in the absence of DWF1. In the rice *br-deficient dwarf2* (*brd2*) mutant, defective in *DWF1*/*DIM*, the production of the bioactive DS has been suggested to explain its mild BR-deficient phenotype (Hong et al. 2005). However, the level of DS was also below the detection limit in the current experiment.

### *DWF4* alters canopy architecture with no negative pleiotropic effects on grain development

The *dwf4-abd* mutant exhibited significantly reduced FLA at anthesis and produced compact spikes with a mild reduction in plant height in the field. Grain size and TGW were unaffected, consistent with the low expression of *DWF4* in developing grains in contrast to *DWF1* expression (Figs. S6 and S7) (Ramírez-González et al. 2018). However, a detailed comparison of BR content and grain structure during development between *dwf1*, *dwf4* and WT would be necessary to inform the lack of effect of *dwf4* on grain size. This organ-specific effect is consistent with earlier studies in rice, where *DWF4* knockout mutants exhibit modified leaf angle without affecting height or grain traits (Sakamoto et al. 2006).

The limited pleiotropic effects of the *dwf4-abd* mutant suggests this gene may be a promising target for breeding wheat ideotypes that are suited for high-density planting. In maize, *lac1*, a *zmdwf4* null mutant, maintains photosynthetic efficiency at high planting densities in contrast to the wild-type, allowing higher yields (Tian et al. 2024). Space limitations meant we were unable to compare canopy cover for the wheat *dwf4* mutant using the Field Scanalyzer system. Although *dwf4* did not produce an increase in TGW compared with Cadenza in the field experiment, it may enable higher yields than the control at high planting densities. Therefore, to test this hypothesis in wheat, it will be necessary to compare yields for *dwf4*-*abd* and *DWF4-NS* genotypes from large plot field experiments under different sowing densities.

Scanning electron microscopy of the adaxial and auricle/collar region of the lamina joint indicated that the leaf blade was more tightly packed around the ligule in *dwf1-abd* and *dwf4-abd* than in Cadenza. This phenotype resembles the wheat *compact plant architecture* (*cpa*) mutant, in which a loss-of-function mutation in *TaSPL8* downregulates genes in the BR and auxin signalling pathways (Liu et al. 2019). Additionally, both *dwf1* and *dwf4* exhibited significantly reduced cell lengths in the auricle region, likely limiting longitudinal elongation and preventing leaves from bending away from the stem. This feature is commonly reported in erect-statured rice and maize BR-related mutants (Feng et al. 2016; Zhao et al. 2013; Zhang et al. 2015; Tian et al. 2019). This reduction in cell lengths caused significant thickening of the entire lamina joint in the mutants compared to Cadenza (Fig. 5). Additionally, in comparison to the wild-type, there were more cell layers in the regions including sclerenchyma adjacent to both the abaxial and adaxial surfaces of the lamina joint in *dwf1* and *dwf4* mutants. Increased sclerenchyma cell layers has been reported for BR mutants of rice and maize, although in these cases only one of the layers was affected, at the abaxial in rice and the adaxial region in maize (Sun et al. 2015; Tian et al. 2019).

Exogenous BL restored seedling leaf angle in *dwf4-abd*, demonstrating that DWF4 contributes to endogenous BR biosynthesis, controlling leaf inclination (Choe et al. 1998). However, BR levels in *dwf4-abd* seedlings were not significantly different from the controls, which was also observed in rice, where the levels of BR intermediates were altered in the *osdwf4-1/osdwf11-4* double mutant but not in either single mutant (Sakamoto et al. 2006). This discrepancy may be due to the tissue-specific expression of *DWF4* within the lamina joint, which was not captured in our whole-seedling analysis. Supporting this possibility, the maize *lac1* mutant exhibits reduced levels of 6-deoxoCT and CS specifically in the ligule region (Tian et al. 2024). Therefore, a more detailed spatial and temporal analysis of BR metabolites in wheat lamina joints will be required to determine whether similar localized variation in BR distribution determines the erect-leaf phenotype of *dwf4-abd*. To further investigate the spatial specificity of *DWF4*, it would be necessary to determine the effect of the *dwf4* mutation on BR content in a broad range of organs.

## Conclusions

Loss-of-function mutations in *DWF1* and *DWF4* both confer an upright canopy architecture in wheat. However, because *DWF1* is the only enzyme catalysing the conversion of 24-MC to CR early in the BR biosynthesis pathway, null mutations also cause reductions in height, tiller number and grain size, pleiotropic effects that will likely restrict the utility of *dwf1* loss-of-function alleles for crop improvement. By contrast, *dwf4* null alleles confer erect-leaf architecture with only a mild reduction in height and no effect on grain development. Therefore, introducing natural or induced allelic variation in *DWF4* may be a promising strategy to breed wheat ideotypes suited to high-density planting conditions.

## Supplementary Information

Below is the link to the electronic supplementary material.

Supplementary file1

## Data Availability

All data generated in this study are provided in the manuscript and supplementary files. Biological materials are available upon request from Stephen Pearce.
