# Supplementary material for: Knocking-out the brassinosteroid biosynthesis genes DWARF1 or DWARF4 produces upright canopy architecture in wheat
Source: Theor Appl Genet. 2026 Oct 24;139(11):313. doi: 10.1007/s00122-026-05418-z (PMC13695666; doi:10.1007/s00122-026-05418-z)
Supplement: Supplementary file 1 [file 122_2026_5418_MOESM1_ESM.docx]

**Knocking-out the brassinosteroid-biosynthesis genes *DWARF1* or *DWARF4* produces upright canopy architecture in wheat**

Manpartik S. Gill, Andrew L. Phillips, Danuše Tarkowská, John Addy, Nicolas Virlet, M. John Foulkes, Darren M. Wells, Jonathan A. Atkinson, Stephen Pearce, Stephen G. Thomas, Peter Hedden

**Additional file 1**

**Figure S1:** *DWF1* and *DWF4* homoeologues and mutant alleles used in this study.

**Figure S2:** Phenotypic characterization of all combinatorial *dwf1* mutants under glasshouse conditions.

**Figure S3:** Phenotypic characterization of all combinatorial *dwf4* mutants under glasshouse conditions.

**Figure S4:** Phenotype of Cadenza and *dwf1-abd* mutant at the tillering and grain filling stages in the field.

**Figure S5:** High throughput phenotyping of *dwf1-abd* mutant under Field Scanalyzer.

**Figure S6:** Expression of *DWF1* genes in wheat variety ‘Azhurnaya’.

**Figure S7:** Expression of *DWF4* and *DWF11* genes in wheat variety ‘Azhurnaya’.

**Table S1:** Homoeologue-specific primers designed to amplify fragments around the deleterious mutations in *TaDWF1* and *TaDWF4* genes.

**Table S2:** KASP primers designed to differentiate the mutant and wild type allele in segregating *DWF1* and *DWF4* populations.

**Table S3:** BR levels (pg/mg DW) in *dwf1-abd* and *dwf4-abd* mutants and controls.

**Figure S1: *DWF1* and *DWF4* homoeologues and mutant alleles used in this study.** (A) *TaDWF1* gene indicating the position of nonsense mutations in the three homoeologues (red arrows) (B) Structure of the TaDWF1 protein showing conserved domains and the predicted TaDWF1 proteins encoded by the mutant alleles. (C) *TaDWF4* gene indicating the position of nonsense mutations in the three homoeologues (red arrows) (D) Structure of the TaDWF4 protein showing conserved domains and the predicted TaDWF4 proteins encoded by the mutant alleles. Position of nonsense mutations identified from the Cadenza TILLING population are indicated. * = stop codon. Produced using Biorender.com.

**Figure S2: Phenotypic characterization of all combinatorial *dwf1* mutants under glasshouse conditions.** (A) Flag Leaf Angle (FLA) at 22 days post anthesis, (B) Final plant height (n = 4), (C) Number of tillers/ plant (n = 4), and (D) Internode lengths (n = 4) of *tadwf1* combinatorial mutants along with Cadenza. *p*-values indicate significant differences between genotypes to *DWF1-NS* (obtained from Fisher’s unprotected LSD test). *** and * indicate significant difference from *DWF1-NS*, *P* < 0.001, and 0.05 > *P* > 0.01, respectively. ns is non-significant.

**Figure S3: Phenotypic characterization of all combinatorial *dwf4* mutants under glasshouse conditions.** (A) Flag Leaf Angle (FLA) at anthesis stage, (B) Final plant height (n = 4), (C) Internode lengths (n = 4), (D) Number of tillers/ plant (n = 4), and (E) Spike length (n = 4) of *tadwf4* combinatorial mutants along with Cadenza. *p*-values indicate significant differences between genotypes to *DWF4-NS* (obtained from Fisher’s unprotected LSD test). * indicate significant difference from *DWF4-NS*, 0.05 > *P* > 0.01, respectively. ns is non-significant.

**Figure S4:** **Phenotype of Cadenza and *dwf1-abd* mutant at the tillering and grain filling stages in the field.** The images show 1 m^2^ plots of Cadenza and the *dwf1-abd* mutant at the tillering and grain filling stages. The row to row and plant to plant spacing was the same in each plot.

**Figure S5: High throughput phenotyping of the *dwf1-abd* mutant under the Field Scanalyzer.** RGB images of *dwf1-abd* (A) and Cadenza (B) at GS69 under the Field Scanalyzer during 2020-21. The left panel shows the original image and the right panel shows the segmented RGB image. RGB images showing highlighted spikes in red in *dwf1-abd* (C) and Cadenza (D) at GS69. These images were used to estimate spike density (number of spikes/m^2^).

**Figure S6:** **Expression of *DWF1* in wheat cultivar ‘Azhurnaya’.** The organ expression profile is shown as transcripts per million (TPM) for the *TaDWF1* homoeologues. The data were obtained from <https://www.wheat-expression.com/>.

**Figure S7: Expression of *DWF4* and *DWF11* in wheat cultivar ‘Azhurnaya’.** The organ expression profiles are shown as transcripts per million (TPM) for *TaDWF4* and *TaDWF11* homoeologues. The data were obtained from <https://www.wheat-expression.com/>.

**Table S1:** **Homoeologue-specific primers designed to amplify fragments around the deleterious mutations in *DWF1* and *DWF4*.** The primer sequences, fragment lengths and Tm (℃) are listed.

| Gene | Forward primer (5' - 3') | Reverse primer (5' - 3') | Amplicon length (bp) | Tm (°C) |
| --- | --- | --- | --- | --- |
| *DWF1-A* | ATGTGGTCCGCCATGAAGTCC | TCGACCTCGAAGTGCCTGACG | 188 | 66 |
| *DWF1-B* | TCAGCTAACATCTCTTTGTACA | TCACGACCTTCTGCACGTTC | 265 | 60 |
| *DWF1-D* | TCAGCTAACATCTCTTTGTACT | TGGGGATGAGCTTGATCTCA | 745 | 60 |
| *DWF4-A* | TCAGTACAGGCATGCGAGCAG | CGCGGACACCACCCCCTTCAT | 660 | 66 |
| *DWF4-B* | TCTCTCTCTCTCCAACCCATCATC | CGCATGCCTGTACTGATGTATA | 403 | 62 |
| *DWF4-D* | GATCTGTGCTAAACTAGACACT | ACGCATATATCTTGCACTGAT | 293 | 62 |

**Table S2:** **KASP primers designed to differentiate between the mutant and wild-type alleles in segregating *DWF1* and *DWF4* populations.** Tail sequences GAAGGTGACCAAGTTCATGCT (FAM) and GAAGGTCGGAGTCAACGGATT (HEX) were added to the 5’ end of the forward primers to differentiate mutant (MT) and wild-type (WT) alleles, respectively. The nucleotide distinguishing wild-type and mutant alleles is in bold.

| Gene | Forward Primer (5' - 3') (MT) | | Forward Primer (5' - 3') (WT) | Common primer (5’ -3’) |
| --- | --- | --- | --- | --- |
| *DWF1-A* | | CACGTTGCGCATGCCGACGGCGAT**T** | CACGTTGCGCATGCCGACGGCGAT**C** | ACCCCAAGAAGGACGGCCTC |
| *DWF1-B* | | ACGAGGATCCATCGGAACT**A** | ACGAGGATCCATCGGAACT**G** | CGACCAAAGAGGAAGAAGGTC |
| *DWF1-D* | | GGAACCCGAGAGTGCCCT**A** | GGAACCCGAGAGTGCCCTG | CGAGTACTCCGACCTCTTCTA |
| *DWF4-A* | | GGCGGCATCCTTGGCAAAT**A** | GGCGGCATCCTTGGCAAAT**G** | CTGAGGAAGTTGAGGGAGATGG |
| *DWF4-B* | | TTCGTCCGGCCGCTCCAC**T** | TTCGTCCGGCCGCTCCAC**C** | CATCCTCCTGGCCCTGCTCA |
| *DWF4-D* | | AACCATCTCTTTGTAGTCTTC**T** | AACCATCTCTTTGTAGTCTTC**C** | ATGGAAATATTGACAGTGAATCCA |

|  | | ***dwf1-abd*** | ***DWF1-NS*** | ***dwf4-abd*** | ***DWF4-NS*** | **Cadenza** | ***p*-value** | **SED** | **LSD at 5%** |
| --- | --- | --- | --- | --- | --- | --- | --- | --- | --- |
|  |  |  |  |  |  |  |  |  |  |
| **BR content (pg/mg DW)** | **CR** | 6.23 ± 0.43**** | 759.05 ± 67.02 | 721.78 ± 61.77* | 811.45 ± 21.46 | 603.61 ± 55.63 | <.001 | 42.165 | 89.872 |
|  | **CN** | 1.27 ± 0.34**** | 16.56 ± 0.78 | 18.99 ± 0.81 | 19.28 ± 0.94 | 17.51 ± 1.84 | <.001 | 0.868 | 1.85 |
|  | **6-oxoCN** | 0.22 ± 0.03*** | 0.82 ± 0.12 | 0.72 ± 0.12 | 0.78 ± 0.19 | 0.79 ± 0.17 | <.001 | 0.112 | 0.239 |
|  | **6-deoxoCT** | 4.11 ± 0.43* | 4.71 ± 0.33 | 4.81 ± 0.12**** | 3.19 ± 0.47 | 3.46 ± 0.10 | <.001 | 0.27 | 0.575 |
|  | **6-deoxoTY** | 40.00 ± 5.20 | 33.41 ± 1.98 | 36.24 ± 3.80**** | 16.09 ± 1.45 | 38.88 ± 5.57 | <.001 | 3.238 | 6.903 |
|  | **TY** | <LOD | | | | |  |  |  |
|  | **CS** | <LOD | | | | |  |  |  |
|  | **BL** | <LOD | | | | |  |  |  |
|  | **DS** | <LOD | | | | |  |  |  |

**Table S3:** **BR levels (pg/mg DW) in *dwf1-abd* and *dwf4-abd* mutants and controls.** Mean values (pg/mg DW) ± SD are shown for the *dwf1-abd* and *dwf4-abd* mutants along with the *DWF1-NS, DWF4-NS* and Cadenza controls at the seedling stage. The data were analysed using one-way ANOVA which yielded *p*-values, SED, and LSD at 5% level of significance. Fisher’s unprotected LSD test was performed for multiple pair-wise comparisons. *P* values *0.05>*P*>0.01, **0.01>*P*>0.001, ****0.0001>*P*>0.00001 (obtained from Fisher’s unprotected test). LOD, limit of detection.
